# Supplementary material for: Palliative care need in the Eastern Mediterranean Region and human resource requirements for effective response
Source: PLOS Glob Public Health. 2023 Nov 3;3(11):e0001980. doi: 10.1371/journal.pgph.0001980 (PMC10624269; doi:10.1371/journal.pgph.0001980)
Supplement: Attachment — Submitted filename: Peer Review - PLOS_PGPH.docx [file pgph.0001980.s001.docx]

Dear authors,

Subject: Palliative Care Need in the Eastern Mediterranean Region and Human Resource Requirements for Effective Response

Thank you for the opportunity to review this manuscript regarding this study on the palliative care need and staffing requirements in the Eastern Mediterranean Region (EMR). Please see the comments for suggested improvements to the manuscript:

**Major Comments:**

Overall, this paper was well-written, and well anchored in the literature. The methods section could benefit from referencing how staffing estimates were calculated (eg. on pg 7, line 144-145, it would be helpful to reference a formula, framework, or instrument used to find the number of FTE required). This would greatly reduce reader confusion on how those values were attained.

**Comments by Section:**

**Introduction:**

- The description around Palliative Care within the Eastern Mediterranean Region was sufficient within the Introduction.
- The context behind why the EMR is the focus of this paper is brought at the very end of the Introduction. It may be helpful to mention the importance of the palliative care needed within this region earlier in the Introduction and further elaborating on the challenges currently in place.
  - Within this region, what percentage of the population is within the EMR system and how often is EMR typically used? Answering this question within the Introduction would allow readers to better understand how frequently the EMR is being utilized and also shed light on the percentage of patient population that may not be frequently receiving healthcare.
- The last few sentences in the Introduction (lines 89-92) may be better suited for the Methods section as they describe what is done in this study.

**Methods:**

- Some concepts within the Methods could be better explained (such as the distributed-opioid oral morphine equivalents (DOME)) to give the reader more context to better understand how the study was conducted.
- Please be more explicit about the data sources used. It is not clear enough from a reproducibility stand point.

**Results:**

- Table 1 and Figure 2 show Pakistan to almost appear as an outlier in the upper and lower bound of symptom days, yet there isn’t really any discussion of trends in this data. If possible, it may be helpful to briefly mention or provide some context for such observations in the results section to improve reader understanding and allow a more comprehensive overview of EMR palliative needs.
- If there is any numerical data on the current state of palliative care in the EMR (eg. current reported number of FTE PC staff in one of the listed countries), it might help to provide this information to compare current staffing with required staffing. This might make the EMR palliative care need/requirements more apparent to readers.
  - I liked the consideration of the FTEs within this paper.

**Discussion:**

- Although this paper is focused on the palliative care needs within the EMR, it would be interesting to draw parallels and insights from other regions that have palliative care needs and describe the current research and interventions attempting to better integrate palliative care in those regions. This may help better contextualize this study within ongoing work in this field in other regions around the world.
- Did the EMR report all patient data, or would some patient data not be included within the EMR? If not all patients are included, it would be worthwhile to include this within the limitations paragraph of the discussion.

**Grammar, Spelling, Punctuation Edits:**

- Pg 10, line 202 “A total of 772 full-time…”. It says “772” FTE doctors needed, but Table 2 below says “722” FTE doctors for doctor-specialists under the column “Afghanistan-LIC”. This seems like a typo (772 ≠ 722) that can be corrected.
- Table 2 - Just for consistency, it might be helpful to capitalize “pc” on the left side of the table
