# Supplementary material for: Palliative care need in the Eastern Mediterranean Region and human resource requirements for effective response
Source: PLOS Glob Public Health. 2023 Nov 3;3(11):e0001980. doi: 10.1371/journal.pgph.0001980 (PMC10624269; doi:10.1371/journal.pgph.0001980)
Supplement: Attachment — Submitted filename: 230924 Response to Reviewers - PC need EMR.docx [file pgph.0001980.s002.docx]

**Palliative Care Need in the Eastern Mediterranean Region and Human Resource Requirements for Effective Response – Manuscript Number: PGPH-D-23-00751**

**List of Changes – Response to Reviewers**

| **Reviewers comments** | **Response to Reviewers** |
| --- | --- |
| **Reviewer #1** |  |
| The author should expand/ explain the challenges with access to Palliative care in the EMR region and the other five WHO regions. The literature review should provide more context to some of the drivers of poor access. | We expanded discussion of the challenges to access to palliative care in the Eastern Mediterranean Region and worldwide, and added key references. |
| define how access to palliative care is defined globally. This will be relevant to comparing against the measure described in the the methodology section line 123 and 124 | We described the ways that palliative care access has been estimated until now. |
| Line 158 – Table 1  It might be helpful to reflect the population size of each of the countries to provide a sense of prevalence of need | We added to the Results the population sizes of the two most populated countries and the two least populated countries in the Region to illustrate the population gradient in need for palliative care.  Table 2 provides the population sizes of the three other countries whose palliative care needs we analyzed in detail.  We feel that these data provide an adequate sense of need. |
| Line 212 Discussion section  The author could expand on how these findings relate with other publications on the same across other WHO regions | We added to the Discussion an explanantion that there are no similar estimates of palliative care human resource needs for any other region or any LMIC. There are only a few estimates from high-income countries of palliative care specialist needs or needs in special circumstances. We also added references. |
|  |  |
| **Reviewer #2** | |
| Authors need to work on solid rationale. The concept sounds but rationale to start the study is weak. Some other statistiscal tool can be used instead of presently used things. | It was difficult for us to know what to do in response to this comment. We respectfully submit that the effort to estimate in detail the burden of suffering in every country in the Eastern Mediterranean Region is of great medical and ethical importance, as is the effort to estimate in detail the human resources needed to respond effectively. Our study uses the methods developed for the Lancet Commission on Palliative Care. The World Health Organization (WHO) approved use of this pioneering method for its Atlas of Palliative Care.  We welcome other investigators to propose other methods to improve these important estimates of human suffering and resources needed to relieve it. But we believe that our methods are currently the state of the art. |
| Result and discussion sections need to more improvements and need technically sound. | We have revised the Results and Discussion sections to make them more clear and to make it easier for future investigators to build on our work. |
| It should be more good if authors prefer to compare UIC rather than lower and middle | We assume the reviewer means “HICs” (high-income countries)?  If so, we would like to point out that we include data from HICs in the region in our study. We do focus on LMICs, and we feel that this focus is justified by the data showing that palliative care is least accessible in LMICs and thus that suffering associated with serious illness remains unrelieved much more often in LMICs than in HICs. |
| Future implications need some more things which will be auspicious for upcoming authors. | We added to each section of the paper clarifications, additional information, and additional references to assist future investigators to build on our work. |
|  |  |
| **Reviewer #3** |  |
| Overall, this paper was well-written, and well anchored in the literature. | Thank you |
| The methods section could benefit from referencing how staffing estimates were calculated (eg. on pg 7, line 144-145, it would be helpful to reference a formula, framework, or instrument used to find the number of FTE required). This would greatly reduce reader confusion on how those values were attained. | We added more detail on how the staffing estimates were made, and we included a reference to the Technical Note and Data Appendix of the LC report where the details of the method were published. |
| The description around Palliative Care within the Eastern Mediterranean Region was sufficient within the Introduction. | Thank you |
| The context behind why the EMR is the focus of this paper is brought at the very end of the Introduction. It may be helpful to mention the importance of the palliative care needed within this region earlier in the Introduction and further elaborating on the challenges currently in place. | Discussion of palliative care need in the EaMReg shifted as suggested to earlier in the Introduction. |
| Within this region, what percentage of the population is within the EMR system and how often is EMR typically used? Answering this question within the Introduction would allow readers to better understand how frequently the EMR is being utilized and also shed light on the percentage of patient population that may not be frequently receiving healthcare. | The reviewer seems to have interpreted “EMR’ to denote “electronic medical record” rather than “Eastern Mediterranean Region” as stated in the Introduction and elsewhere. To prevent this misinterpretation, we have changed the abbreviation “EMR” to “EaMReg”. |
| The last few sentences in the Introduction (lines 89-92) may be better suited for the Methods section as they describe what is done in this study. | We shifted these sentences to the beginning of the Methods section as suggested. |
| Some concepts within the Methods could be better explained (such as the distributed-opioid oral morphine equivalents (DOME)) to give the reader more context to better understand how the study was conducted. | We improved the explanation of distributed-opioid oral morphine equivalents (DOME) improved.  We also added a better explanation of how staffing (FTE) estimates were calculated. |
| Please be more explicit about the data sources used. It is not clear enough from a reproducibility stand point. | We added to the Methods section more detail on the data sources:   - WHO Global Health Estimates (GHE) - International Narcotics Control Board data on distributed-opioid oral morphine equivalents (DOME) - Literature review - Panel of ten experienced palliative care physicians from LMICs in Africa, Asia and the Americas |
| Table 1 and Figure 2 show Pakistan to almost appear as an outlier in the upper and lower bound of symptom days, yet there isn’t really any discussion of trends in this data. If possible, it may be helpful to briefly mention or provide some context for such observations in the results section to improve reader understanding and allow a more comprehensive overview of EMR palliative needs. | We provided context on differences in palliative care need between countries, including populations of the largest and smallest countries in the region. |
| If there is any numerical data on the current state of palliative care in the EMR (eg. current reported number of FTE PC staff in one of the listed countries), it might help to provide this information to compare current staffing with required staffing. This might make the EMR palliative care need/requirements more apparent to readers. | We added a sentence explaining that we know of no previous estimates of human resource requirements to enable universal access to palliative care for all in need in the Eastern Mediterranean Region, even in the best overviews of palliative care in the region. We added references to these overviews. |
| I liked the consideration of the FTEs within this paper. | Thank you. We agree that this is crucial information that has not existed anywhere until now. |
| Although this paper is focused on the palliative care needs within the EMR, it would be interesting to draw parallels and insights from other regions that have palliative care needs and describe the current research and interventions attempting to better integrate palliative care in those regions. This may help better contextualize this study within ongoing work in this field in other regions around the world. | We added sentences comparing palliative care needs and availability in other regions and by country income level.  Our paper focuses on estimates of palliative care needs, and of the resources needed to respond, in the Eastern Mediterranean Region, not on current efforts to implement or integrate palliative care services. We feel that discussion of such efforts is beyond the scope of our paper and would require a very different and large study. |
| Did the EMR report all patient data, or would some patient data not be included within the EMR? If not all patients are included, it would be worthwhile to include this within the limitations paragraph of the discussion. | Again, the reviewer seems to have interpreted “EMR’ to denote “electronic medical record” rather than “Eastern Mediterranean Region” as stated in the Introduction and elsewhere. To prevent this misinterpretation, we have changed the abbreviation “EMR” to “EaMReg”. |
| Pg 10, line 202 “A total of 772 full-time…”. It says “772” FTE doctors needed, but Table 2 below says “722” FTE doctors for doctor-specialists under the column “Afghanistan-LIC”. This seems like a typo (772 ≠ 722) that can be corrected. | Typo corrected. The correct number is 722. |
| Table 2 - Just for consistency, it might be helpful to capitalize “pc” on the left side of the table | PC capitalized as suggested. |
